# Effects of High-Temperature, High-Pressure, and Ultrasonic Treatment on the Physicochemical Properties and Structure of Soluble Dietary Fibers of Millet Bran

**DOI:** 10.3389/fnut.2021.820715

**Published:** 2022-01-18

**Authors:** Chunhong Wei, Yunfei Ge, Dezhi Liu, Shuting Zhao, Mingzhi Wei, Junchen Jiliu, Xin Hu, Zhigang Quan, Yunjiao Wu, Youtao Su, Yifei Wang, Longkui Cao

**Affiliations:** ^1^College of Food Science, Heilongjiang Bayi Agricultural University, Daqing, China; ^2^Department of Marine Food Science and Technology, Gangneung-Wonju National University, Gangneung, South Korea; ^3^National Coarse Cereals Engineering Research Center, Heilongjiang Bayi Agricultural University, Daqing, China

**Keywords:** millet bran, physical modification, soluble dietary fiber, physicochemical properties, structure

## Abstract

**Objectives:**

The effects of high-temperature, high-pressure, and ultrasonic treatment on the physicochemical properties and structure of soluble dietary fibers in millet bran were studied to provide a comprehensive reference for the utilization of millet bran.

**Methods:**

Different physical methods were used to treat millet bran dietary fibers, and their microstructures and Fourier-transform infrared spectra before and after modification were compared. The physicochemical properties (water-holding capacity, swelling capacity, oil-holding capacity, fat-binding capacity, cation exchange capacity), total antioxidant capacity, and thermal characteristics were also analyzed.

**Results:**

There were no significant changes in the chemical groups of millet bran's soluble dietary fibers after modification, but cracks appeared on the surface of the fibers and the structure became loose and porous. Fiber agglomeration was observed, as well as improved thermal stability. After modification, the water-holding capacity, swelling capacity, oil-holding capacity, fat-binding capacity, and cation exchange capacity of millet bran were improved. When compared to the original soluble dietary fibers, ultrasound-treated fibers showed the most substantial improvement in all four capabilities, with increases of 140, 50, 78.1, 65.7, and 37.8%, respectively, compared with the original soluble dietary fibers (*P* < 0.05). The total antioxidant capacity of the ultrasound-treated fibers was found to be higher than those of the fibers that underwent the other three treatments (*P* < 0.05).

**Conclusions:**

The physicochemical qualities and structural characteristics of the soluble dietary fibers in millet bran are affected by all three physical modification methods; however, the physicochemical properties of the ultrasound-treated fibers are most significantly improved.

## Introduction

Millet, a type of cereal grain, belongs to the Gramineae family and originated in the Yellow River Basin of China. It has been a very important category of food crops throughout history, and it was included in the sixth category of food crops in the list of worldwide agricultural products in 2015 (1). In terms of cultivation and breeding, millet has the advantages of drought tolerance, high vitality, short fertility period, and abundant varieties, and is mainly grown in the arid and semi-arid regions of North China, such as the famous peach blossom rice produced in the Hebei region, millet from the northern Shaanxi region, etc. Statistically, China has the largest total annual production of millet, accounting for ~4/5 of the world's total output of millet (2). Millet bran is one of the by-products of millet processing that primarily consists of millet hulls. Nowadays, this millet by-product is mostly used to make fodder, and is rarely used in food production. It is primarily due to the rough taste of millet bran, which does not meet popular dietary standards. However, removing millet bran during processing results in significant nutrient loss from millet. Millet bran contains more vitamin B, C, and E than millet kernels, and the bran accounts for 18% of the millet's fiber content (3), making it a very important source of dietary fibers (DF), and some researchers have shown that millet bran dietary fiber modified by combined ultrasonic-microwave treatment inhibits alpha-glucosidase activity more effectively (4). DF is defined as “a plant edible tissue or comparable carbohydrate that is resistant to digestion and absorption in the human small intestine and is entirely or partially fermented in the large intestine” according to the US definition (5). At present, the classification of dietary fibers is mainly based on the difference in solubility. Accordingly, DF are divided into two categories: soluble DF (SDF) and insoluble DF (IDF), among which SDF has a high functional nutritional value. In terms of intestinal disease prevention, since SDF is fermented in the intestine, it is conducive to the growth and reproduction of locally beneficial microorganisms and further for regulation of the intestinal flora. This helps prevent intestinal diseases (6) such as intestinal cancer. Moreover, consumption of the non-toxic, harmless, and beneficial SDF can have a certain regulatory effect on human blood sugar and insulin level and plays a role in the alleviation of diabetes (7). Many other studies have indicated that the presence of SDF in the human body can significantly affect the metabolism and transformation of cholesterol, which can be beneficial in the treatment of cardiac diseases and atherosclerosis (8). Application of water-soluble dietary fibers is now mainly achieved by means of modification to reduce waste and increase its economic benefits.

Chemical, biological, physical, and combination changes (9) are the most common ways to modify dietary fiber, according to developments in research on the subject. Chemical modification mainly involves the application of acidic or alkaline chemical reagents to treat the fibers. Wei (10) used apple dietary fibers as raw material and found that the yield of apple SDF increased after acidic and alkaline treatments. The water-holding capacity (WHC) and swelling capacity (SC) of the modified apple SDF were greatly improved. Biological modification usually refers to treatment using enzymes; the types of enzymes commonly used for this purpose are cellulase, hemicellulase, etc. Qian et al. (11) hydrolyzed the rice bran dietary fiber with cellulase and found that the ability of modified rice bran dietary fiber to remove DPPH (1,1-diphenyl-2-picrylhydrazyl) etc. was enhanced. The research results of Wang et al. (12) showed that the ultrafine crushing made the SDF powder particles distributed densely and agglomerated; the combination of superfine grinding and ultrasonic-microwave could improve the SDF cation exchange capacity (CEC) of millet significantly, and improve the ability to combine with selenium. Physical modification is mainly the treatment using mechanical force, including high-pressure homogenization, extrusion expansion, high-temperature, and so on. Su et al. (13) treated citrus peel with high-pressure homogenization, and found that the physicochemical properties of citrus dietary fiber were greatly improved. Gao et al. (14) found that extrusion expansion treated carrot peels showed an increase in the content of SDF. The oil-holding capacity (OHC) and WHC of the treated carrot peels were also dramatically increased. Wang et al. (15) used bamboo shoots as raw material and cooked them under a high temperature. They discovered that the treatment changed a portion of the IDF in bamboo shoots into SDF, and the physiochemical properties of bamboo shoots dietary fiber were significantly improved in terms of WHC, SC, and OHC. Shen and Cao (16) applied ultrasonic-microwave synergistic treatment to black bean peels to study the optimal processing conditions for extracting SDF from them.

However, reported studies on the modification of millet bran in China have mainly been focused on optimizing the extraction process of raw dietary fiber and increasing the yield (17). Few research have looked at how millet bran's SDF can be altered, resulting in changes in its physicochemical qualities and structure. There are few studies on the effects of raw material alterations on the structural and physicochemical properties of SDF. Furthermore, oats, bamboo shoots, wheat, and other common raw materials for SDF research are oats, bamboo shoots, wheat, and so on, although millet bran research is uncommon. To the best of our knowledge, the application of the three physical alterations to millet bran SDF and the comparison of the physicochemical properties and structure of SDF before and after modification have never been reported earlier. In this study, the modification of millet bran SDF was achieved by high-temperature, high-pressure, and ultrasound treatment. Scanning electron microscopy (SEM), Fourier-transform infrared (FTIR) spectroscopy, and differential scanning calorimetry (DSC) were used to determine the apparent structure, functional group structure, thermal stability, WHC, SC, OHC, FBC, and CEC of SDF before and after modification. This research could provide a complete reference for millet bran use as well as a theoretical foundation for developing functional millet bran products that match national dietary needs.

## Materials and Methods

### Experimental Materials

Millet bran were purchased from Tuogu Millet Factory (Daqing, China), Neutral protease, heat-stable α- amylase, amyloglucosidase were purchased from Sigma Corporation (Beijing, China), 95% ethanol (AR Grade) were purchased from tianjin Yongsheng Fine Chemical Co (Tianjing, China), Soybean oil were purchased from Changchun Jiayu Grain and Oil Co (Changchun China).

### Instruments and Equipment

TGL16B Benchtop Centrifuge Shanghai Shuangxu Electronics Co (Shanghai, China); GDE-CSF6 VELP Dietary Fiber Analyzer Beijing Ying Sheng Heng Tai Technology Co (Beijing, China); DK-S24 Thermostatic Water Bath Shanghai Baxin Instrument Factory (Shanghai, China); MLS-3781L-PC Autoclave Shanghai BaJiu Industrial Co (Shanghai, China); Ultrasonic Medicine Processor Jining Jinbite Electronics Liability Co (Jinan, China); SU8020 Scanning Electron Microscope Shanghai Shuangxu Electronics Co (Shanghai, China); Nicolet 6700 Fourier Transform Infrared Spectrometer Suzhou Sato Precision Instruments Co (Suzhou, China); DSC25 Differential Scanning Calorimeter Artbridge Technology (HK) Ltd (Beijing, China); DGG-9070A Electric Heating Thermostatic Blast Drying Oven Shanghai Senshin Testing Instruments Co Beijing; Specord-200 UV-Visible Spectrophotometer Analytik Jena (Beijing, China).

### Preparation of SDF From Millet Bran

Defatted millet bran (20 g) was mixed with 1,000 mL of distilled water and 1,000 mL of phosphate buffer solution (PBS). Heat-stable α- amylase was first added to allow enzymatic digestion of millet bran in a water bath at 95°C for 20 min. Neutral protease was then added to the mixture for further enzymatic digestion in a water bath at 60°C for 30 min, followed by the addition of amyloglucosidase and incubation in a water bath at 60°C for 30 min. The enzymes were deactivated, and the mixture was condensed to 1/4 of the original volume. The condensed solution was mixed with 95% ethanol at 1:4 (v/v) and left in the refrigerator at 4°C for 15 h to allow precipitation. The precipitate was centrifuged at 4,000 rpm for 20 min. And the final precipitate was collected and freeze-dried at −108°C to obtain millet bran SDF (18).

### Three Modifying Treatments of Millet Bran SDF

#### High-Temperature Treatment

Millet bran SDF (2 g) obtained as described in section of preparation of SDF from millet bran, and was weighed, placed in a conical flask, and thoroughly mixed with 60 mL of distilled water. The mixture was transferred to a high-temperature cooker and processed at 125°C for 50 min before being centrifuged at 5,000 rpm for 15 min. The supernatant was collected and mixed with 95% ethanol at 1:4 (v/v) ratio to allow precipitation. The modified millet bran SDF was obtained by centrifugation at 4,000 rpm for 20 min, followed by collecting the pellet, and drying at 60°C (19).

#### High-Pressure Treatment

Millet bran SDF (2 g) obtained as described in section of preparation of SDF from millet bran, and was weighed and placed in a conical flask and mixed well with 60 mL of distilled water. The mixture was transferred to an autoclave and processed at 0.1 MPa for a certain time. After cooling, the mixture was centrifuged at 5,000 rpm for 15 min. The supernatant was collected and mixed with 95% ethanol at 1:4 (v/v) ratio for ethanol precipitation. The modified millet bran SDF was obtained by centrifugation at 4,000 rpm for 20 min, followed by collecting the pellet, and drying at 60°C (20).

### Ultrasonic Treatment

Millet bran SDF (2 g) obtained as described in section of preparation of SDF from millet bran, and was weighed and placed in a conical flask and mixed well with 60 mL of distilled water. The mixture was treated with ultrasound at a power of 50 W for 1 h. The modified millet bran SDF was obtained by drying the treated mixture at 60°C until the moisture content was about 6% (21).

### Determination of the Surface Microstructure of Millet Bran SDF

Zongcai's method was used to determine the surface microstructure of millet bran SDF (22). Two milligram of millet bran SDF was spread out on a double-sided conductive tape, mounted on the operating stage, and then the surface was coated with gold by ion sputtering. The coated millet bran SDF was observed and analyzed using a scanning electron microscope, and images of the clear regions were obtained at magnifications of 20,000 × and 50,000×.

### Determination of SDF FTIR Spectra of Millet Bran

FTIR spectroscopy was carried out according to Qian's method, which involved weighing and transferring 2 mg of millet bran SDF into a mortar. 200 mg of KBr powder (SDF: KBr = 1:100) was added to the millet bran SDF (23). The mixture was then crushed, ground, and pelleted using a vacuum tablet press. Finally, the spectra of the mixed sample tablets were determined by a FTIR spectrometer with a scanning range of 4,000–400 cm^−1^.

### Determination of Physicochemical Properties of Millet Bran SDF

#### Determination of WHC

Referring to the method described by Chen et al. (24), 1 g of millet bran SDF was weighed and transferred to a 50 mL centrifuge tube and soaked in 20 mL of distilled water for 12 h. It was then centrifuged at 4,000 rpm for 25 min, and the pellet was collected and weighed. WHC was calculated using the following equation:


$$\begin{array}{l} W=\frac{m_{1}-m_{2}}{m_{3}} \end{array}$$

where W was the WHC (g/g), m_1_ was the mass of the SDF along with the centrifuge tube after soaking (g), m_2_ was the mass of the centrifuge tube (g), and m_3_ was the dry mass of the SDF (g).

#### Determination of SC

The SC was determined using the method described by Robertson (25). Millet bran SDF (0.25 g) was weighed and transferred to a 10 mL graduated test tube and mixed with 5 mL of distilled water. The mixture was stirred, defoamed, and left to stand still for 12 h. SC was then calculated with the following equation:


$$\begin{array}{l} E=\frac{V_{1}-\;V_{2}}{m} \end{array}$$

where E was the SC (mL/g), V_1_ was the volume of the swollen sample (mL), V_2_ was the volume of the dry sample (mL), and m was the dry mass of the sample (g).

#### Determination of OHC

OHC was determined using the method described by Chau and Huang (26) with slight modifications. Millet bran SDF (0.5 g) was weighed and transferred to a 50 mL centrifuge tube and mixed with 4 g of soybean oil. The tube containing samples was sealed and incubated in a 37°C water bath for 4 h. The mixture was centrifuged at 4,000 rpm for 15 min to obtain the pellet, and OHC was calculated using the following equation:


$$\begin{array}{l} O=\frac{m_{1}-{\;m}_{2}}{m_{2}} \end{array}$$

where O was the OHC (g/g), m_1_ was the mass of the oil-treated SDF (g), and m_2_ was the original mass of the SDF (g).

#### Determination of FBC

The FBC was determined using the method described by Li et al. (27) with slight modifications. Millet bran SDF (4 g) was weighed and transferred to a 50 mL centrifuge tube. 20 mL of soybean oil was added to the sample, and the sample was stirred every 5 min for a total of 30 min. The mixture was centrifuged at 1,600 rpm for 25 min. Free fat was removed by centrifugation and the FBC could be calculated as the amount of fat bound to 1 g of SDF.

#### Determination of CEC

Refer to Ling et al. (28) method for determining the CEC of millet bran SDF with various treatments: 0.5 g SDF in an Erlenmeyer flask, 30 mL 0.1 mol/L hydrochloric acid solution, keep 12 h at room temperature, filter and wash with distilled water to neutralize, transfer the processed sample to 100 mL 5 % NaCl solution, mix with a magnetic stirrer, and titrate with 0.1 mol/L sodium hydroxide solution. The reaction indicator was a 0.5% phenolphthalein-ethanol solution, and the titration end point was the solution's non-fading for 3 min after changing color. Simultaneously, a blank experiment was carried out. The following was the formula for calculating cation exchange capacity:


$$\begin{array}{l} N=\left(\left(V1-V0\right)\times 0.1\right)/m \end{array}$$

Where N was the CEC(mmol/g), V1 was the volume of sodium hydroxide solution consumed in titrating the sample/mL, V0 was the volume of sodium hydroxide solution consumed in titrating the blank sample/mL, and m was the mass of the sample/g.

### Determination of *in vitro* Antioxidant Activity of Millet Bran SDF

Total antioxidant capacity (TAOC) kit was used to determine the antioxidant activity of millet bran SDF (29). The specific procedure was performed per instructions in triplicates. The absorbance of millet bran SDF was measured at a wavelength of 520 nm. The TAOC was then calculated using the following equation:


$$\begin{array}{l} T\;\left(U/mL\right)=\frac{OD_{sample}-\;OD_{control}\;}{0.01}÷30\times \frac{\;V_{total}\;}{V_{sample}}\times A \end{array}$$

where T (U/mL) was the TAOC, OD_sample_ was the absorbance of the SDF samples, OD_control_ was the absorbance the control, V_total_ (mL) was the total volume of the reaction solution, V_sample_ (mL) was the original sampling volume, and A was the times of dilution of the sample before testing.

### Determination of the Thermal Properties of Millet Bran SDF

The determination of the thermal stability of millet bran SDF was performed using the method described by Wang et al. (30) with slight modifications. The sample (3–5 g) was weighed and transferred into an alumina tray. The parameters for the DSC (27) differential scanning calorimeter were selected as follows: the temperature increase rate was set to 10°C/min, nitrogen was selected as the atmosphere, the flow rate was set to 50 mL/min, and the temperature was varied in the range from 0 to 250°C. The calorimeter was then switched on and preheated, followed by sample testing.

### Statistical Analysis

Microsoft Excel (Redmond, WA, USA) and SPSS software (IBM Corp, Armonk, NY, USA) were used for statistical analysis. Origin software (OriginLab Corporation, Northampton, MA, USA) was used for image processing. All data was collected in triplicate and the average value was used for analysis.

## Results

### Surface Microstructure of Millet Bran SDF Before and After the Three Modifications

**Figure 1:**
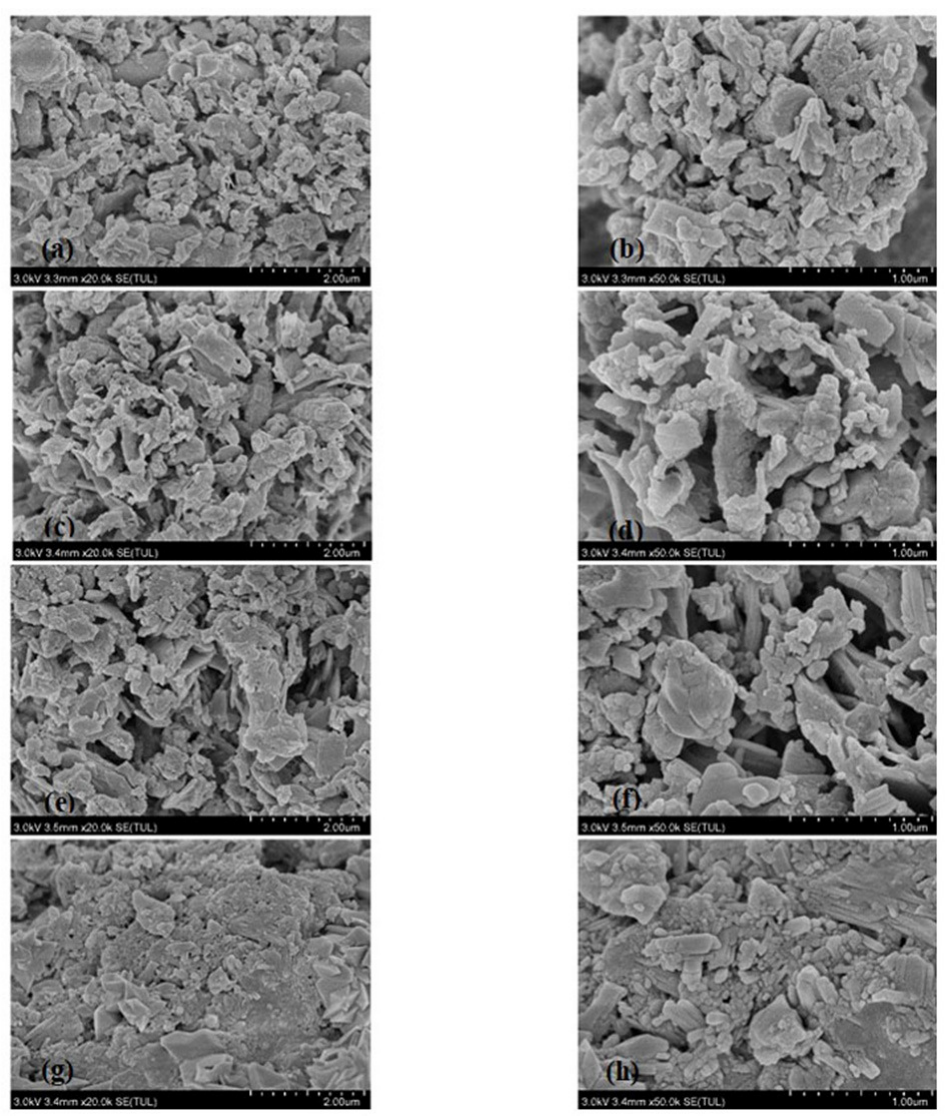
SEM images of SDF of millet bran before and after three modifications **(a)** untreated SDF at 20,000 ×, **(b)** untreated SDF at 50,000 ×, **(c)** high-temperature-cooked SDF at 20,000 ×, **(d)** high-temperature-cooked SDF at 50,000 ×, **(e)** high-pressure-treated SDF at 20,000 ×, **(f)** high-pressure-treated SDF at 20,000 ×, **(g)** ultrasound-treated SDF at 20,000 ×, **(h)** ultrasound-treated SDF at 50,000 ×.

The micromorphology of millet bran SDF before and after the three modifications was shown in Figure 1. Figures 1a,b show that the overall structure of unmodified millet bran SDF was denser and closely packed. Figures 1c,d, the overall structure of millet bran SDF appeared to become loose and porous. Figures 1e,f showed that the compact structure of millet bran SDF changed to a loose honeycomb-like mesh structure with large gaps after high-pressure treatment, in Figures 1g,h, the surface structure of millet bran SDF was relatively denser with higher degree of adhesion after ultrasonic treatment.

### FTIR Spectroscopy of Millet Bran SDF Before and After the Three Modifications

**Figure 2:**
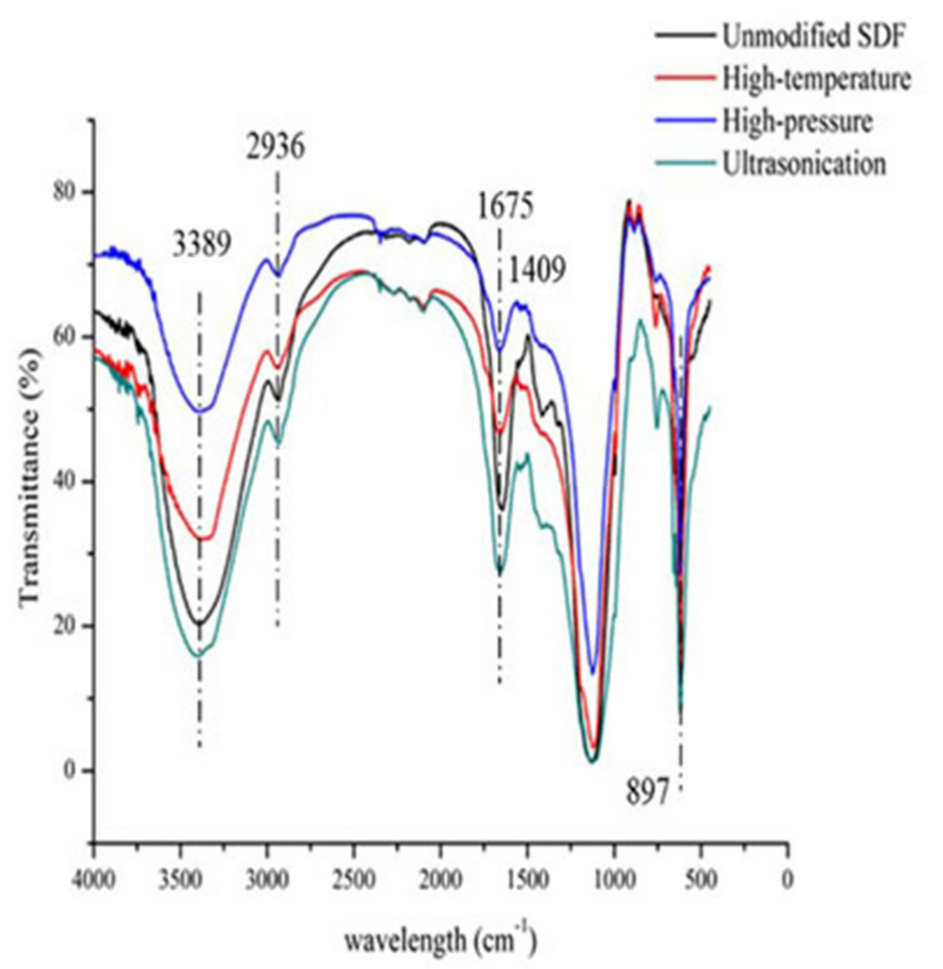
Infrared spectra of millet bran SDF before and after three modifications.

The FTIR spectra of millet bran SDF before and after the three modifications were shown in Figure 2. It was found that the spectral distributions of all the four samples were similar. There was no appearance of new peaks, indicating that the physical modification did not change the content of functional groups or their ways of binding in millet bran SDF.

### WHC, SC, OHC, FBC and CEC of Millet Bran SDF Before and After the Three Modifications

As shown in Table 1, the WHC of millet bran SDF before modification was 0.42 g/g, SC was 0.80 mL/g, OHC was 2.20 g/g, FBC was 1.81 g/mL and CEC was 0.45 mmol/g. All five capacities were increased after high-temperature and high-pressure, and ultrasonic treatment. All five capacities were significantly increased by 140, 50, 78.1, 65.7, and 37.8%, respectively, after ultrasonic treatment (*P* < 0.05).

**Table 1:** Determination of physicochemical properties of millet bran SDF before and after three modifications.

**Property**	**Unmodified SDF**	**High-temperature**	**High-pressure**	**Ultrasonication**
WHC (g/g)	0.42 ± 0.10^c^	0.55 ± 0.12^bc^	0.71 ± 0.13^b^	1.01 ± 0.11^a^
SC (mL/g)	0.80 ± 0.07^c^	0.91 ± 0.08^bc^	1.00 ± 0.10^b^	1.20 ± 0.08^a^
OHC (g/g)	2.20 ± 0.80^c^	2.72 ± 0.11^bc^	3.24 ± 0.30^a^	3.92 ± 0.48^a^
FBC (g/mL)	1.81 ± 0.06^c^	2.00 ± 0.12^c^	2.35 ± 0.15^b^	3.00 ± 0.20^a^
CEC(mmol/g)	0.45 ± 0.03^c^	0.52 ± 0.02^b^	0.57 ± 0.01^ab^	0.62 ± 0.02^a^

*Note: The same superscripts in different columns of the peer group indicate no significant difference, while different superscripts indicate that the physicochemical properties of the samples before and after modification are significantly different at the 0.05 level*.

### *In-vitro* Antioxidant Activity of Millet Bran SDF Before and After the Three Modifications

**Figure 3:**
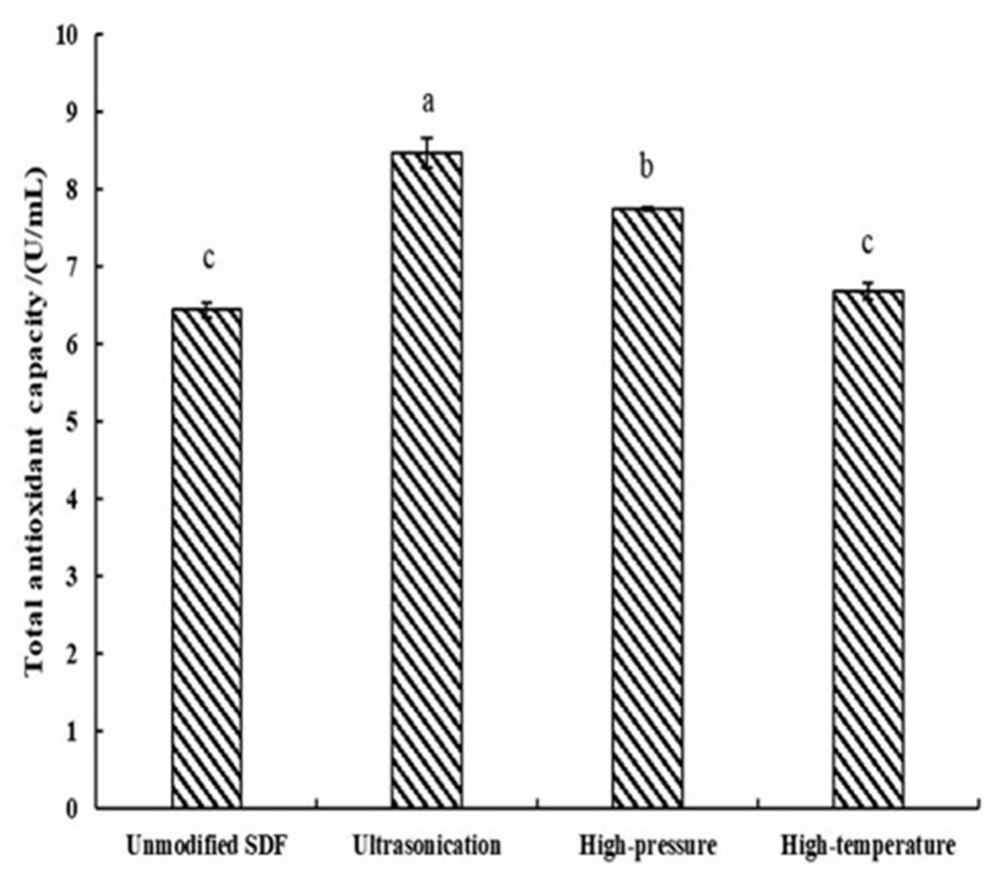
TAOC of SDF of Millet bran before and after three modifications. The same superscripts indicate no significant difference, while different superscripts indicate that the physicochemical properties of the samples before and after modification are significantly different at the 0.05 level.

As demonstrated in Figure 3, the TAOC of the samples treated with high pressure and ultrasound significantly increased compared to the unmodified samples by 20.2 and 31.5% (*P* < 0.05), respectively.

### Thermal Properties of Millet Bran SDF Before and After the Three Modifications

The mass fractions of millet bran SDF under different temperatures before and after the three modifications were shown in Table 2. At 200°C, the mass fraction of unmodified millet bran SDF was 92.38%, and that of high-pressure-modified millet bran SDF was higher (94.76%). At 300°C, the mass fraction of unmodified millet bran SDF was 82.60%, and that of ultrasonically treated millet bran SDF was higher (85.75%).

**Table 2:** Mass fractions of Millet bran SDF before and after three modifications.

**Sample**	**Mass fractions @200°C**	**Mass fractions @300°C**
Unmodified SDF	92.38%	82.60%
High-temperature cooking	93.74%	84.40%
High pressure	94.76%	85.37%
Ultrasonication	94.60%	85.75%

## Discussion

In summary, Scanning electron microscopy showed that the surface of unmodified millet dietary fiber was uneven and appears irregularly agglomerated, presenting large but ununiform particles (31), the overall structure of millet bran SDF appeared to become loose and porous with a rough surface after high-temperature (32), the compact structure of millet bran SDF changed to a loose honeycomb-like mesh structure with large gaps after high-pressure treatment. A part of the agglomerated large particles were also transformed into aggregated small particles after modification, which may be a result of the breaking of molecular bonds of dietary fibers due to the forces (shear force, collision force, etc.) generated during high-pressure treatment (33), the surface structure of millet bran SDF was relatively denser with higher degree of adhesion after ultrasonic treatment, which could be mainly because of the ultrasonication-induced increase of hydroxyl groups and hydrogen bonds within SDF. This theory was further confirmed with an increase in peak area around 3,400 cm^−1^ in the FTIR spectrum (Figure 2). The surface appeared to be unevenly wrinkled, could be due to ultrasonication disrupting the structure. The rupture of air bubbles created by ultrasonication could give rise to micro-jets that impact the surface structure and generate gaps (34). In a nutshell, the modified SDF surface developed numerous cracks, and the structure became loose and porous. It could be a result of the absence of hemicellulose and lignin due to cross-linking during the modifying treatments, which leaded to an increase in porosity as well as the pore size. It would ultimately play an important role in the improvement of the physicochemical properties of SDF (35).

As evident in Figure 2, the FTIR spectra of millet bran SDF modified by high-temperature, high-pressure, and ultrasound treatment were analyzed and compared with the spectrum of the untreated SDF. The difference in absorption intensities at the corresponding wavelengths indicated the changes in physicochemical properties of SDF (36). A broad absorption peak corresponding to the intramolecular O-H stretching vibration was observed at 3,389 cm^−1^, which may be a result of the formation of hydrogen bonds between the hydroxyl groups within the SDF molecules. The peak indicating υ(C-H) stretching vibration band of polysaccharide methyl and methylene appeared at 2,936 cm^−1^. The modifications weakened the intensity of this peak, indicating that the cracks in the millet bran SDF became larger, potentially leading to an increase in the modified sample's OHC (37). These results were further consistent with the findings of SEM and the results from the OHC tests as the modified millet bran SDF showed significantly higher OHC. As a possible consequence of asymmetric C=O stretching vibrations, a distinct sharp peak appears at 1,675 cm^−1^, indicating the presumable presence of glyoxalate in the sample (29). A small peak at 1,408 cm^−1^ was attributed to σ(C-H) stretching vibrations, and some diminished peaks around 1,608 cm^−1^ may be related to C=O or σ(-OH) stretching vibrations (38). A strong absorption peak at 1,124 cm^−1^ was attributed to the characteristic C-O stretching vibrations and O-H deformation vibrations of polysaccharides (39). The moderate-intensity peak at 897 cm^−1^ indicated the existence of mannosidic bonds in SDF (40). Overall, the intensity of the characteristic absorption peaks of the infrared spectra of millet bran SDF modified by high-temperature, high-pressure, and ultrasound treatment varied. But the shapes and positions of the characteristic peaks did not change significantly. It can be concluded that these three modifications did not have a prominent effect on the functional groups of millet bran SDF (32).

The results of the physicochemical property tests of the three modified millet bran SDFs before and after modifications were shown in Table 1. WHC, SC, OHC, FBC and CEC are important indicators of the physicochemical properties of dietary fiber. When the five capacities are higher, the dietary fiber has a better physiological activity (41), and the functional properties of the dietary fiber can be utilized to the maximum extent. All five capacities were increased after high-temperature and high-pressure, and ultrasonic treatment, the increases may be due to the disruption of the dietary fiber structure by the strong forces such as the shear force generated by the mechanical shock during the ultrasonic treatment. During the treatment, the meshed structure of the dietary fibers was disrupted, some of the large particles were broken down into smaller ones, and the structural porosity and the specific surface area increased. All of these changes exposed the hydrophilic groups of SDF as water binding sites, as well as an increase in exposed lipophilic groups, which eventually led to the enhancement in WHC, OHC, SC, FBC and CEC (42). The results of these tests were in agreement with the results of SEM and FTIR. Overall, the WHC, SC, OHC, FBC and CEC of millet bran SDF modified by high-temperature, high-pressure treatment, and ultrasound treatment were increased.

The TAOCs of millet bran SDF before and after the three modifications were shown in Figure 3. The level of TAOC is an indicator of the electron-providing capacity of the samples (43). the TAOCs of millet bran SDF after high-temperature, high-pressure, and ultrasonic treatment were all increased. The increase is due to a change in the glyoxalate content of the dietary fiber as a result of the modification (44). This result was in accordance with the FTIR results, which showed a strong absorption peak at a wavelength of 1,675 cm^−1^. Thus, the TAOC of millet bran SDF was increased after high-temperature, high-pressure, and ultrasonic modifications.

**Figure 4:**
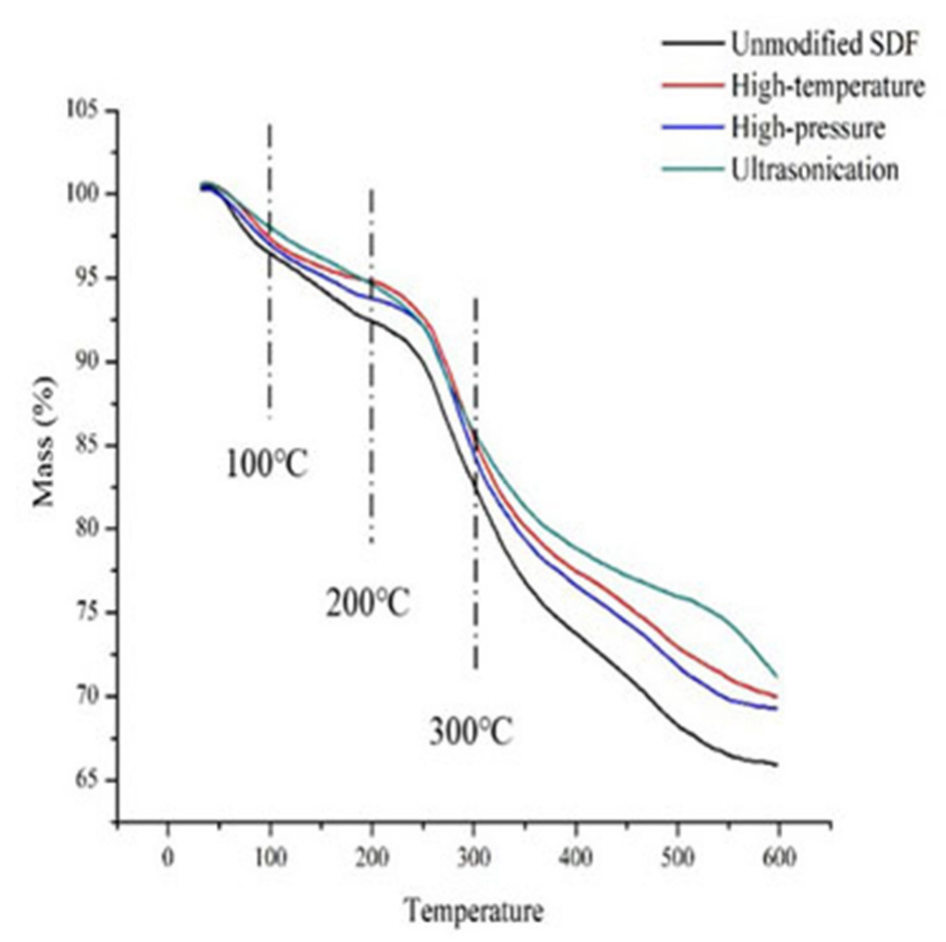
Thermal analysis of SDF of millet bran before and after three modifications.

The thermal properties of millet bran SDF before and after the three modifications were shown in Figure 4. The millet bran SDF thermogravimetric curves could be divided into three stages: 30–100, 200–300, and 450–600°C. The first stage was primarily concerned with the loss of water content in millet bran SDF. The weight loss at this stage is predominantly because of the evaporation of free water and water of crystallization from millet bran SDF under increased temperature (37). The second stage was mainly the macromolecular decomposition. The highest weight loss of millet bran SDF happens at this stage, owing to the gradual depolymerization of macromolecules since the increase in temperature can lead to the decomposition of cellulose and hemicellulose. The third stage was the carbonization stage where the weight loss of millet bran SDF gradually slowed down. When reached this stage, most of the millet bran SDF components have already been decomposed, leaving a little residual material to be decomposed into carbon and ash. The substances that require higher temperatures to decompose were generally considered to have better thermal stability (45). As a result, it is possible to conclude that the thermal stability of millet bran SDF was improved after all three modifications (46), with ultrasonic modification providing the greatest improvement to thermal stability of millet bran SDF.

## Conclusion

In this paper, the SDF of millet bran was modified with three treatments, and their physicochemical properties and structural changes were analyzed before and after the modifications. After the high-temperature, high-pressure treatment, and ultrasonic treatments, the OHC, SC, OHC, FBC, CEC, and TAOC were generally enhanced compared with unmodified millet bran SDF. The ultrasound-treated millet bran SDF showed the greatest improvement. The results of FTIR, SEM, and DSC thermal analysis showed that in the modified millet bran SDF, but the intensity of absorption peaks changed, surface folds were loosened, porosity increased, and TAOC and thermal stability were improved. Thus, the high-temperature, high-pressure treatment, and ultrasonic treatments effectively improved the physicochemical properties and structural characteristics of millet bran SDF, providing a scientific reference for using it as an addition to functional food.

## Data Availability Statement

The original contributions presented in the study are included in the article/supplementary material, further inquiries can be directed to the corresponding author/s.

## Author Contributions

CW: conceptualization, investigation, and editing. YG: writing-original draft, methodology, and writing—review. DL: investigation, validation, and formal analysis. SZ: methodology and writing -review and editing. MW: investigation. JJ: writing—review and editing. XH, ZQ, YWu, YS, and YWa: validation. LC: conceptualization, methodology, supervision, writing—review and editing, and funding acquisition. All authors have read and agreed to the published version of the manuscript.

## Funding

This research was supported by the National Key R&D Porgram of China (2018YFE0206300), Advantageous and Characteristic Discipline Program of Heilongjiang Province Department of Education ([2018]NO.4), Quality improvement and deep processing position in Heilongjiang Province Coarse Cereals Modern Agricultural Industrial Technology Collaborative Innovation System, Construction Project of Engineering Research Center for Processing and Utilization of Grain By-products of the Ministry of Education, Heilongjiang Bayi Agricultural University School Cultivation Project (2041080010).

## Conflict of Interest

The authors declare that the research was conducted in the absence of any commercial or financial relationships that could be construed as a potential conflict of interest.

## Publisher's Note

All claims expressed in this article are solely those of the authors and do not necessarily represent those of their affiliated organizations, or those of the publisher, the editors and the reviewers. Any product that may be evaluated in this article, or claim that may be made by its manufacturer, is not guaranteed or endorsed by the publisher.
